# Supplementary material for: Morphing of liquid crystal surfaces by emergent collectivity
Source: Nat Commun. 2019 Aug 5;10:3501. doi: 10.1038/s41467-019-11501-5 (PMC6683186; doi:10.1038/s41467-019-11501-5)
Supplement: Supplementary file 1 — Supplementary Information [file 41467_2019_11501_MOESM1_ESM.pdf]

Supplementary Information for:

Morphing of liquid crystal surfaces by emergent collectivity

Hanne M. van der Kooij[1,2], *Slav A. Semerdzhiev*[1,2], *Jesse Buijs*[1],
Dirk J. Broer[3,4], *Danqing Liu*[3,4] *and Joris Sprakel*[1*]

[1]*Physical Chemistry and Soft Matter, Wageningen University & Research, Stippeneng 4, 6708 WE Wageningen, The Netherlands*

[2]*Dutch Polymer Institute (DPI), P.O. Box 902, 5600 AX Eindhoven, The Netherlands*

[3]*Stimuli-responsive Functional Materials and Devices, Department of Chemical Engineering and Chemistry, Eindhoven University of Technology, 5612 AE, Eindhoven, The Netherlands*
[4]*Institute for Complex Molecular Systems, Eindhoven University of Technology, 5600 MB, Eindhoven, The Netherlands*

[*]Correspondence to: joris.sprakel@wur.nl

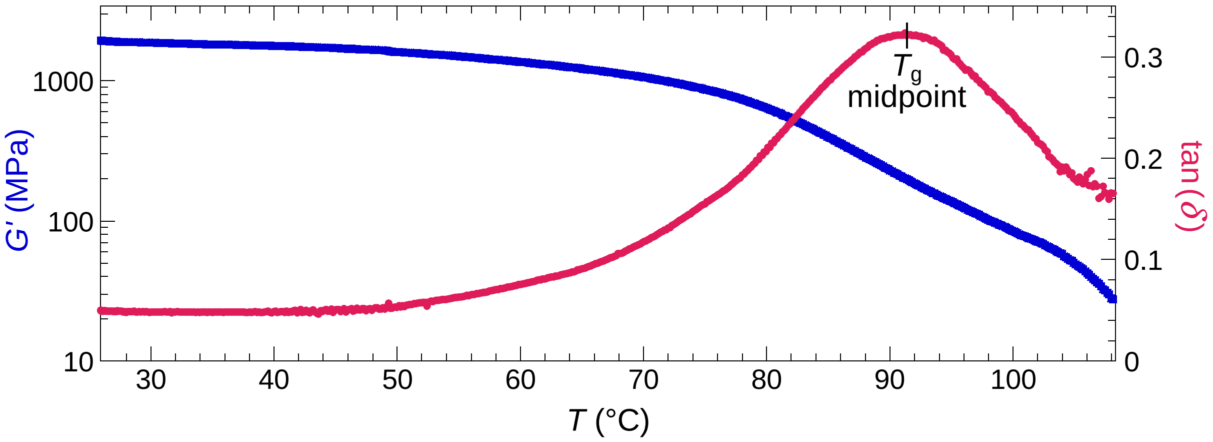

Supplementary Figure 1. Thermomechanical properties. Storage modulus G′ (■) and loss tangent tan(δ) (●) versus temperature for a free-standing, homeotropic LCN film, measured using dynamic mechanical thermal analysis (DMTA) (Q800, TA Instruments, USA). The glass transition is quite broad, ranging from ~60 °C to >110 °C, and centred around 91 °C (peak of loss tangent). The experiment is conducted in tensile mode at a heating rate of 3 °C min^{-1} and oscillation frequency of 1 Hz, with the stretching direction perpendicular to the nematic director.

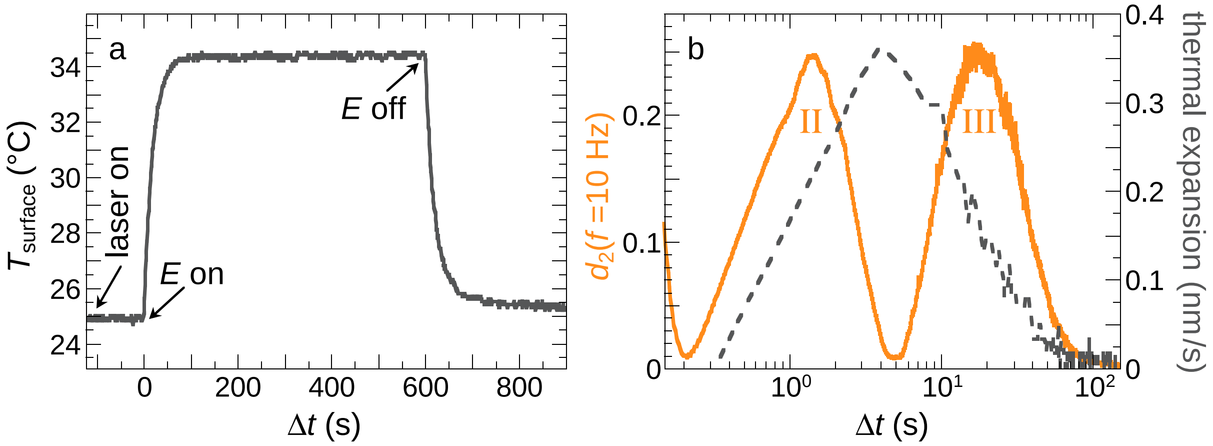

Supplementary Figure 2. Electrothermal effects. **(a)** Changes in the surface temperature of a homeotropic LCN actuated at 900 kHz and 70 V, measured using an infrared sensor. Dielectric heating causes a rise of 9.5 °C within 80 s after switching on the field (at Δt = 0 s), followed by a plateau in which the heat generation and exchange with the surroundings are in equilibrium. The kinetics of cooling after switching off the field (at Δt = 600 s) are very similar. Turning on the laser (at Δt = −100 s) results in negligible heating since the sample absorbs virtually no 532-nm light. Throughout the experiment, the sample temperature remains far below the glass transition range. **(b)** Network deformations (—, left ordinate) and electrothermal expansion rate (- -, right ordinate) computed from **(a)** assuming a thermal expansion coefficient[1] of $3 \cdot 10^{-4}$ K^{-1}. The cumulative electrothermal expansion is ~8 nm. Clearly, dielectric heating does not underlie the three dynamic stages, as the time scales are incompatible. Further evidence is found in the large mismatch of frequencies: the three stages are characterized by frequencies >10 Hz, yet the thermal expansion in $^1/_{10}$ s is <0.04 nm i.e. below the detection limit of LSI. The measured motility thus cannot result from electrothermal effects.

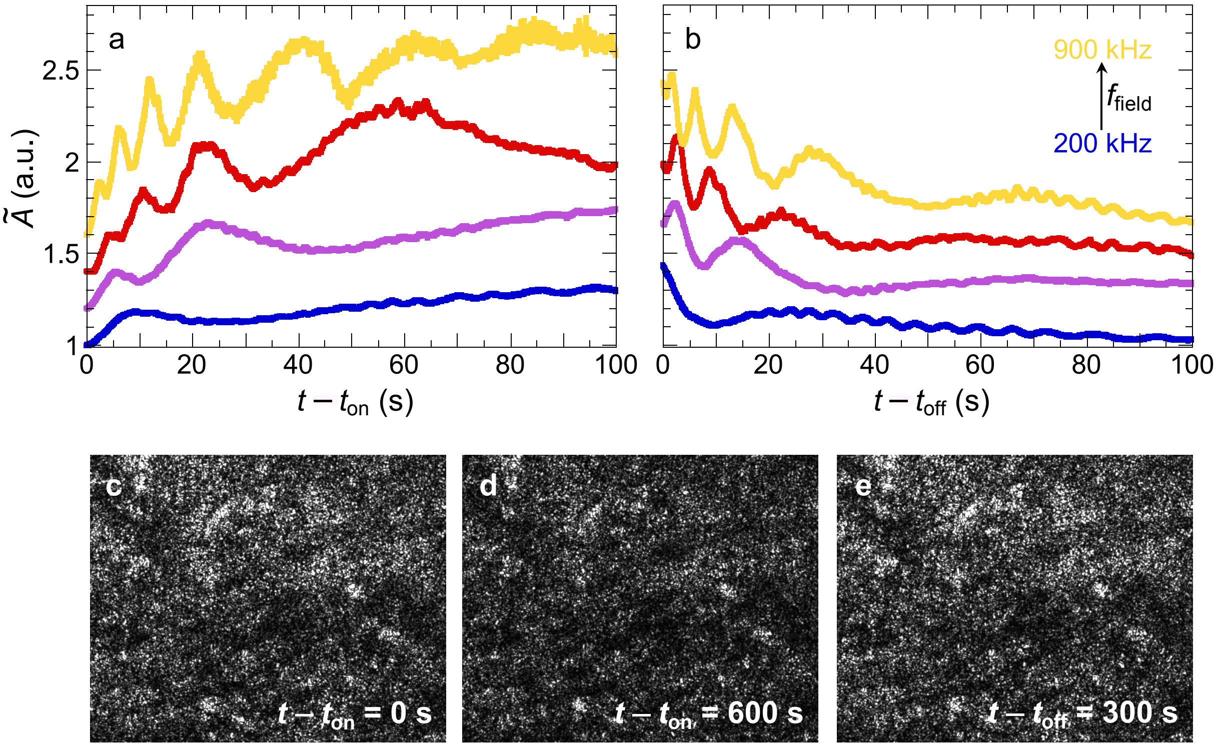

Supplementary Figure 3. Kinetics and reversibility of surface deformations. (a–b) Zooms of Fig. 3 of the main text on linear time axes, to highlight the decrease in undulation frequency over time. Note that the last 500 s of **(a)** and the last 200 s of **(b)** are omitted for clarity. **(c–e)** Speckle patterns corresponding to different times in the 200 kHz on–off cycle: **(c)** before field switch-on, **(d)** 600 s after field switch-on, and **(e)** 300 s after field switch-off. The speckle patterns cover 0.57 mm^2 surface area. The field-induced expansion is manifest as an overall decrease in speckle intensity from **(c)** to **(d)**, which is fully reversed after switching off the field **(e)**. Not only the average scattering intensity is largely recovered, but also the initial speckle pattern, implying that surface morphing does not lead to significant rearrangement or displacement of the TiO_2 particles. Note that at higher field frequencies, the speckle changes are more pronounced and somewhat less reversible.

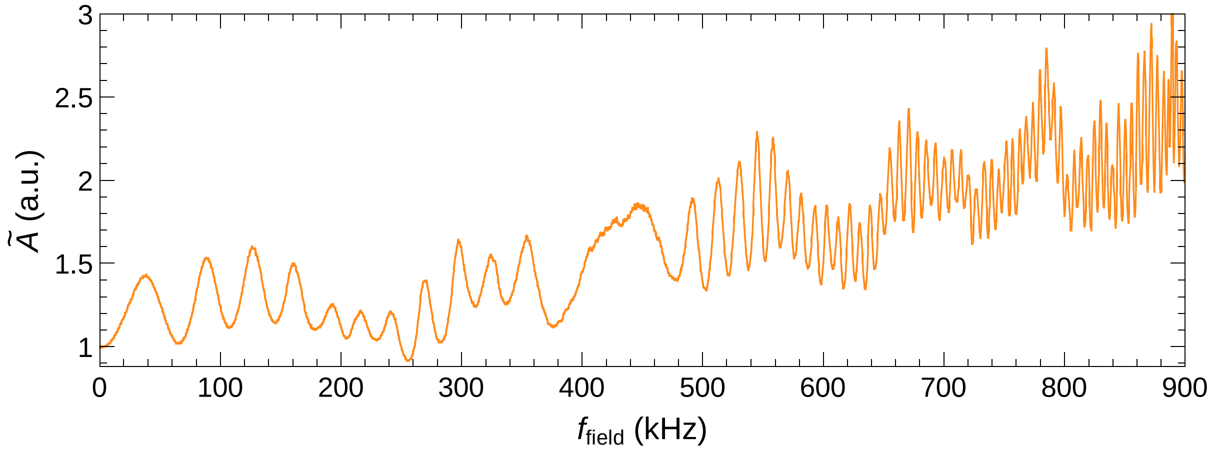

Supplementary Figure 4. Frequency dependence of surface deformations. Change in surface area of a homeotropic LCN during a field frequency sweep from 0 to 900 kHz at 3 kHz s^{-1}. The surface expansion grows with the driving frequency and exhibits undulations with decreasing period, as the dielectric interactions between the field and LCN keep changing during the sweep. The speckle fluctuations are averaged over 250 μm^2 surface.

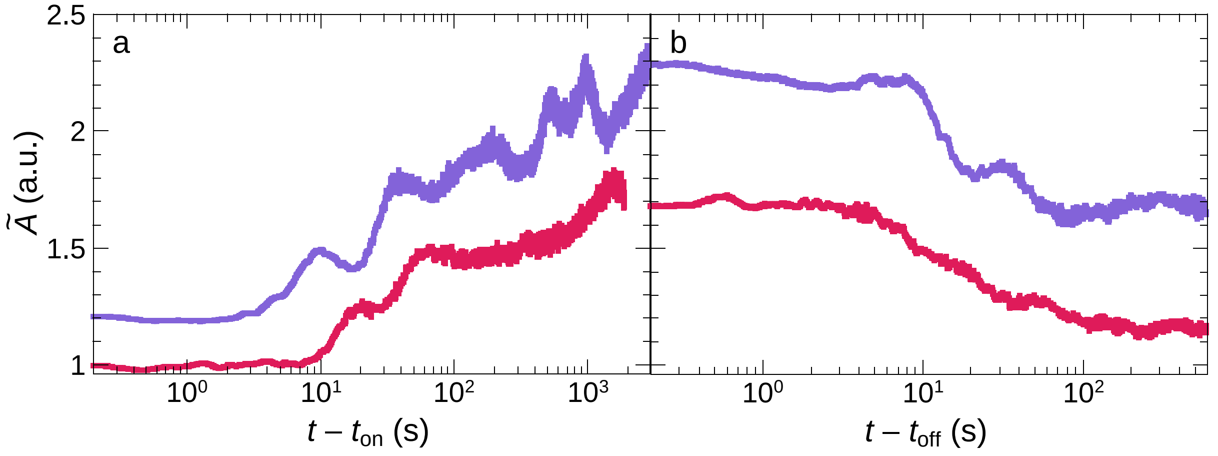

Supplementary Figure 5. Temperature dependent kinetics of surface deformations. Change in surface area of a homeotropic LCN upon switching the field on **(a)** and off **(b)** at temperatures of 45 °C (•) and 65 °C (•). For clarity, the upper curve is offset vertically by 0.2. Some elastic ringing is observed, yet the extent is considerably smaller than at 25 °C (see Fig. 3 • of the main text). Particularly at 65 °C, where the LCN is in its glass transition regime, the oscillations almost vanish. These results confirm that network elasticity is at the origin of the surface undulations.

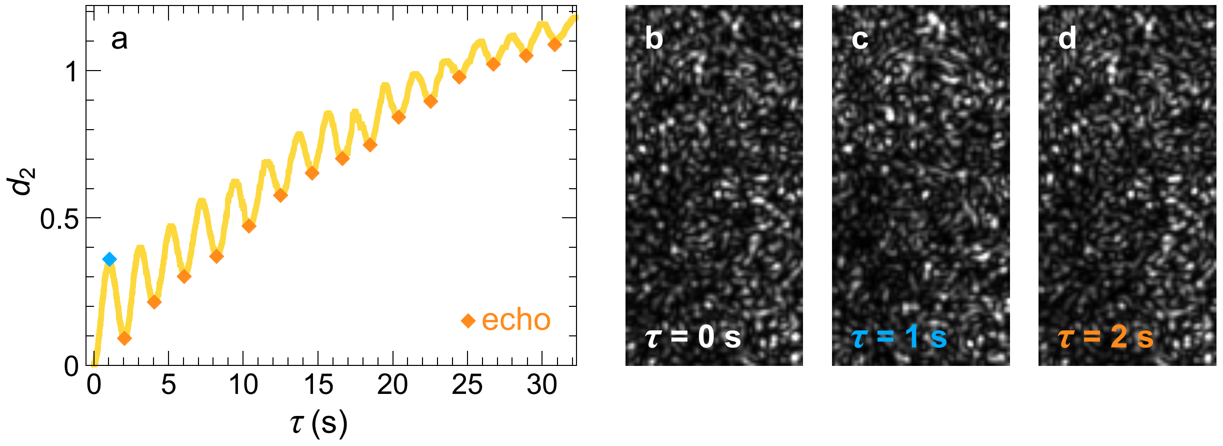

Supplementary Figure 6. Speckle echoes by elastic ringing. (**a**) Intensity structure function $d_2(\tau)$ identical to the inset in Fig. 3b of the main text, displaying distinct echoes (orange diamonds). At these echoes, the speckle pattern reverts to an earlier pattern, reflecting elastic recoil of the surface. Indeed, the pattern at $\tau = 0$ s (**b**) is almost identical to that at $\tau = 2$ s (**d**). By contrast, between echoes the surface shows an overshoot, causing the speckle pattern to transiently change considerably. Pattern (**c**) at $\tau = 1$ s clearly deviates from both (**b**) and (**d**). These features are characteristic of elastic ringing. The oscillations of $d_2(\tau)$ are indeed quite unique, since usually d_2 increases monotonically with increasing lag time τ. The speckle patterns cover 0.015 mm^2 surface area.

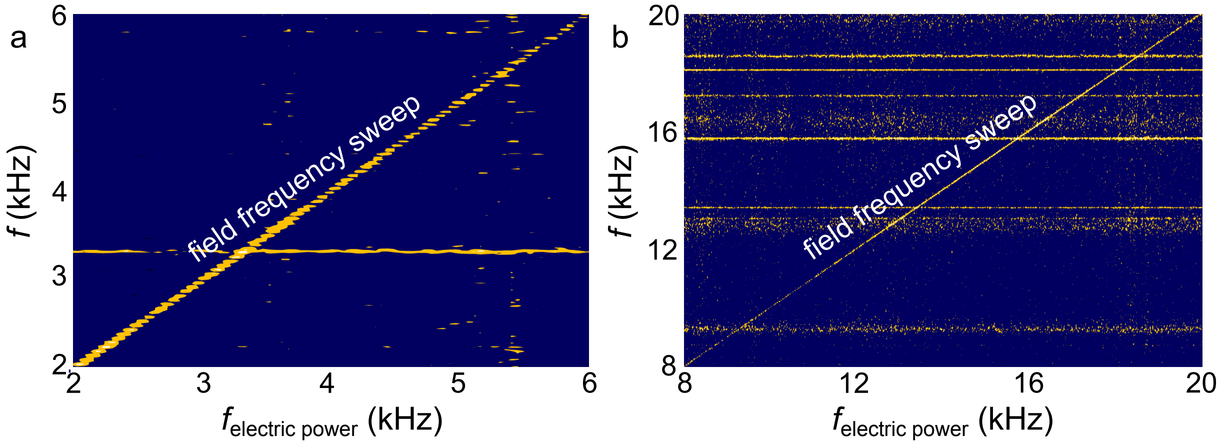

Supplementary Figure 7. Detecting the driving field frequency. Spectrally resolved dynamics (vertical axis) during linear sweeps of the driving frequency (horizontal axis), from 2 to 6 kHz (**a**) and from 8 to 20 kHz (**b**). Each vertical strip represents one power spectrum of the temporal speckle intensity, averaged over 0.0030 mm^2 surface. For clarity, the low-frequency noise is excluded. The horizontal lines correspond to electrical artefacts. Although these driving frequencies are too low to elicit significant surface deformation, pronounced diagonal signals are visible, corresponding to minute dielectric interactions between the field and molecular dipoles. We note that the horizontal axes do not represent f_{field} i.e. the frequency of the electric current I and voltage V, but rather the frequency of the electric power P which is twice as high. Since $P(t) = I(t)V(t)$, it indeed follows that for an AC field, $f_P = 2f_I = 2f_V \equiv 2f_{\text{field}}$ for all possible phase angles between current and voltage. Clearly, it is the electric power which dictates the material response. In view of consistency with previous work, we adhere to f_{field} in the rest of the text.

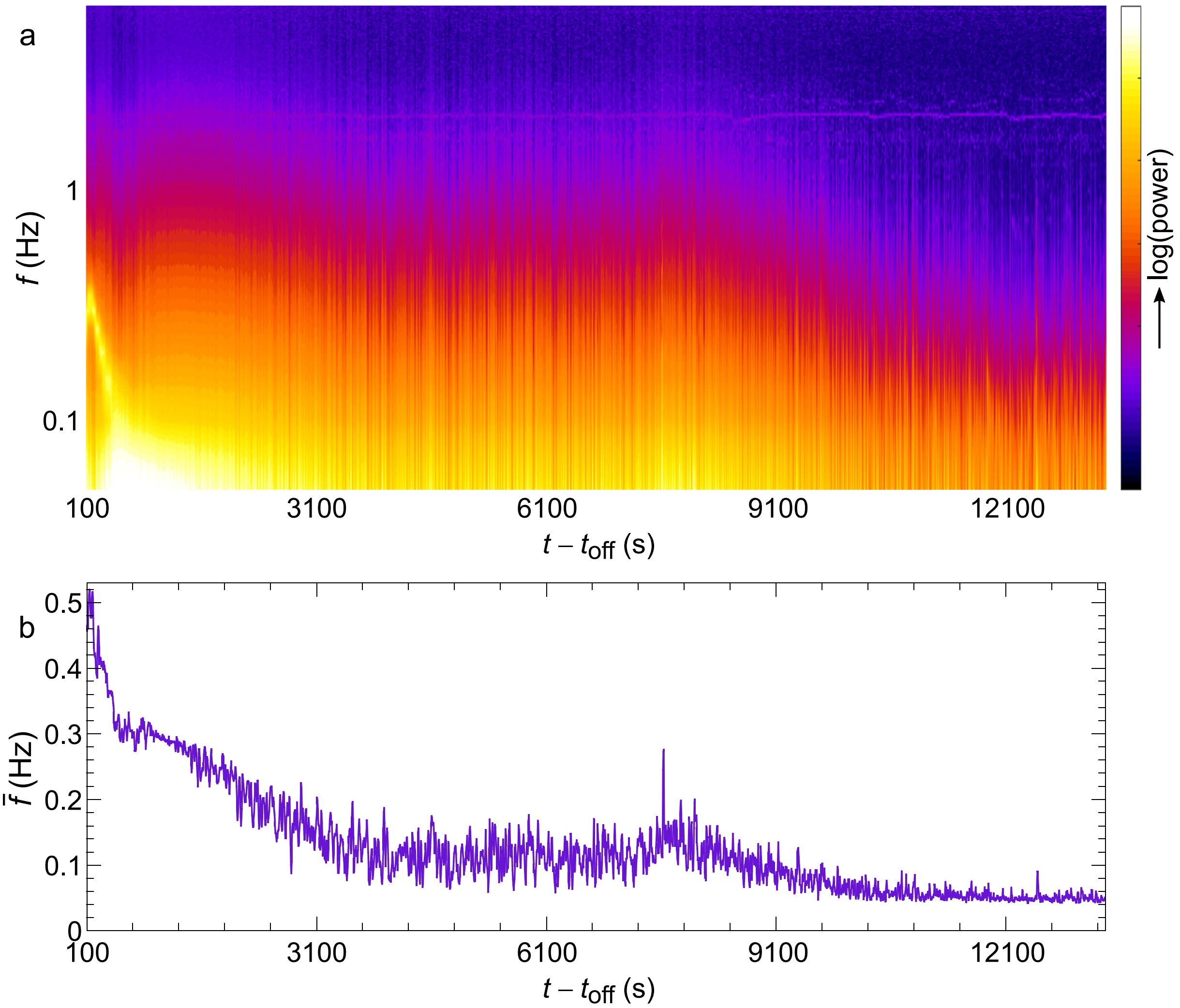

Supplementary Figure 8. Long-term relaxation dynamics. (**a**) Spectrogram of the surface motility of a homeotropic LCN after switching off the field. Each vertical strip represents one power spectrum of the temporal speckle intensity, averaged over 0.57 mm^2 surface. Since the network is glassy, complete relaxation takes hours and is characterized by low frequencies (note the different f-axis compared to Fig. 4a of the main text). (**b**) Mean frequency of the spectrogram in (**a**), computed as $\bar{f} = \sum_{i=1}^{n} f_i P_i$, to highlight the gradual slowing down of the network dynamics in time. The f-axis is logarithmic in (**a**) yet linear in (**b**).

Supplementary Note 1: Fourier-transform laser speckle imaging

The following Matlab routine is used to compute 1D power spectra and 2D spectrograms of speckle intensity fluctuations. First, for each time step, a set of images is loaded into memory. The total number of time steps and number of images per step are tailored to the question at hand and the frame rate employed. The set of images is normalized on a pixel-by-pixel basis by subtracting the time-average intensity from the temporal intensity signal, so that the intensity fluctuates around zero. The power spectrum is subsequently obtained by taking the squared modulus (i.e. absolute value) of the output of the Matlab fft function. The result is a 3D matrix of power values as a function of the two spatial dimensions x and y and frequencies f, which is spatially averaged over all pixels to yield a single power spectrum. This algorithm is iterated over consecutive time steps to produce a power spectrogram.

The highest frequency that can be resolved using Fourier transform is half of the sampling rate – called the Nyquist frequency[2] – which in LSI is half of the camera frame rate. The lowest frequency is determined by the number of images in a set, implying that the frequency range can be extended to lower values at the cost of time resolution, or vice versa the time resolution can be improved at the expense of frequency scope.

The power spectrum and $d_2(\tau)$ are closely related. Specifically, the power spectrum and the electric field correlation function $g_1(\tau)$ are a Fourier pair, which means that the two can be interconverted using the (inverse) Fourier transform[3]. g_1 is directly related to d_2 via: $g_1(\tau) = \sqrt{1 - d_2(\tau)/2\beta}$. Here, β is a numerical constant that accounts for the number of speckles detected per pixel[4].

Supplementary References

[1] Liu, D., Bastiaansen, C. W. M., den Toonder, J .M. J. & Broer, D. J. Light-induced formation of dynamic and permanent surface topologies in chiral-nematic polymer networks. *Macromolecules* **45**, 8005–8012 (2012).

[2] Bergland, G. D. A guided tour of the fast Fourier transform. *IEEE Spectrum* **6**, 41–52 (1969).

[3] Lu, W. & Vaswani, N. The Wiener–Khinchin theorem for non-wide sense stationary random processes. Preprint at https://arxiv.org/abs/0904.0602 (2009).

[4] van der Kooij, H. M., Fokkink, R., van der Gucht, J. & Sprakel, J. Quantitative imaging of heterogeneous dynamics in drying and aging paints. *Sci. Rep.* **6**, 34383 (2016).
